# Tanshinone IIA Downregulates HMGB1 and TLR4 Expression in a Spinal Nerve Ligation Model of Neuropathic Pain

**DOI:** 10.1155/2014/639563

**Published:** 2014-07-10

**Authors:** Yu-Qing Ma, Yi-Rong Chen, Yu-Fang Leng, Zhi-Wei Wu

**Affiliations:** ^1^Department of Anesthesiology, First Hospital of Lanzhou University, Lanzhou, Gansu 730000, China; ^2^Department of Urology, People's Hospital of Gansu Province, Lanzhou, Gansu 730000, China; ^3^Research Center of Molecular Biology Laboratory, Gansu University of Traditional Chinese Medicine, Lanzhou, Gansu 730000, China

## Abstract

Fifty-four Sprague-Dawley rats weighing 200~240 g were randomly divided into sham-operated group (sham group), vehicle-treated SNL group (model group), and Tan IIA-treated SNL group (Tan IIA group). Tan IIA was administered intraperitoneally to rats in the Tan IIA-treated group at a dose of 30 mg/kg daily for 14 days after SNL surgery. Paw withdrawal mechanical thresholds (PWTs) and paw withdrawal thermal latencies (PWLs) were measured. High-mobility group box 1 (HMGB1) and Toll-like Receptor 4 (TLR4) mRNA and protein expression in the spinal cord were measured. Tumour necrosis factor alpha (TNF-*α*), interleukin-1 beta (IL-1*β*), and interleukin-10 (IL-10) in the spinal cord were measured, too. Both the mechanical and heat pain thresholds were significantly decreased. After Tan IIA treatment, HMGB1, and TLR4 mRNA and protein levels, the expression of TNF-*α* and IF-1*β* was reduced significantly. In conclusion, Tanshinone IIA reversed SNL-induced thermal hyperalgesia and mechanical allodynia and downregulated HMGB1 and TLR4 levels and inhibited the HMGB1-TLR4 pathway. Tanshinone IIA inhibited TNF-*α* and IL-1*β* expression but not IF-10 expression in the spinal cords of SNL rats. These results indicate that Tanshinone IIA inhibited SNL-induced neuropathic pain via multiple effects, and targeting the HMGB1-TLR4 pathway could serve as the basis of new antinociceptive agents.

## 1. Background

Neuropathic pain is a chronic disorder that is a consequence of a lesion or disease affecting the somatosensory system [1]. It includes nervous system injury and chronic persistent alterations in pain sensitivity. Chronic pain often responds poorly to NSAIDs and opioids. Therefore, novel target molecules are eagerly anticipated for improving treatment of neuropathic pain.

Numerous studies have revealed that spinal inflammation and the immune response play an important role in neuropathic pain. Nerve injury-induced neuropathic pain is impaired after deletion or inhibition of Toll-Like Receptor 4 (TLR4) [2]. TLR4 is related to immune and inflammatory diseases. Once activated by endogenous or exogenous ligands, TLR4 induces the massive production of early proinflammatory cytokines such as TNF-*α* and IL-1*β* [3, 4]. High expression levels of inflammatory cytokines such as TNF-*α* and IL-1*β* are related to neuropathic pain [5]. Recent evidence has demonstrated that high-mobility group box 1 (HMGB1) can induce neuropathic pain in experimental models. As a proinflammatory mediator, HMGB1 may be released from sensory nerve tissue. It leads to the release of proinflammatory cytokines such as TNF-*α* [6] and contributes to the development of neuropathic pain after nerve injury [7, 8]. The roles of HMGB1 and TLR4 in neuropathic pain are unknown, and more studies are required to confirm whether the HMGB1-TLR4 interaction is directly involved in the pathogenesis of neuropathic pain.

No effective therapy for neuropathic pain has been developed [9, 10]. Tanshinone IIA (Tan IIA) is one of the major active components of the root of* Salvia miltiorrhiza* Bunge. Tan IIA has a similar structure to 17-beta estradiol (E2) and produces anti-inflammatory effects via inhibition of the inflammatory cytokines IL-1*β*, IL-6, and TNF-*α* [11]. The effect of Tan IIA on chronic pain is not known. More studies are required to confirm whether Tan IIA produces protective effects to alleviate hyperpathia in SNL-induced neuropathic pain, and whether Tan IIA downregulates HMGB1 and TLR4 expression in the spinal cord is not yet clear.

## 2. Methods

### 2.1. Animals and Surgical Procedures

Male Sprague-Dawley (SD) SPF rats (weighing 200~240 g) were provided by the Animal Research Centre of Gansu University of Traditional Chinese Medicine (TCM). The rats were housed individually in cages, under a 12-hour light/dark cycle at 20~25°C, with free access to food and water. The experimental rats were allowed to acclimatise to the facility for a week prior to surgery. All experimental procedures were conducted in accordance with the guidelines of the Institutional Animal Care and Use Committee of Gansu University of TCM. All efforts were undertaken to minimise the number of animals used and their discomfort. Fifty-four rats were randomly divided into 3 groups: a sham-operated group (sham group), a vehicle-treated SNL group (model group), and a Tan IIA-treated SNL group (Tan IIA group). Each group was divided into subgroups (*n* = 6 in each) according to the time of sacrifice: 3 days, 7 days, and 14 days.

Tan IIA has a large dose range of 1 mg/kg to 50 mg/kg. Different doses within this range have been used [12–15]. Our preliminary experiments demonstrated that TLR4 and HMGB1 gene and protein levels in the spinal cord were attenuated significantly at doses of 30 mg/kg and 50 mg/kg Tan IIA but not at 10 mg/kg Tan IIA in 12 rats (data not shown). No significant difference between the effective doses was observed. However, 30 mg/kg was selected for further experiments because 50 mg/kg Tan IIA exhibited a greater number of side effects. Tan IIA (30 mg/kg, i.p., Shanghai number 1 Biochemical Pharmaceutical Company, China, Batch number 130408) was injected daily for 14 days after surgery. Sham-operated rats received sterile water for injection as a control. The same dose of sterile water was administered to animals in the vehicle-treated group.

A lumbar (L) 5 spinal nerve ligation (L5 SNL) model was used as the neuropathic pain model. All of the rats were placed under anaesthesia with chloral hydrate (300 mg/kg, i.p.) and subjected to the SNL model. After incision of the skin, the L5 spinal nerve was carefully isolated and tightly ligated with 6~0 silk sutures and was cut at 5 mm distal to the knotting as described by Kim and Chung [16]. In sham-operated rats, the L5 nerve was exposed in the same manner but not ligated.

### 2.2. Behavioural Tests

According to Kim and Chung [16], L5 SNL model rats have been well established as a neuropathic pain model that display hyperalgesia without phenomena, such as paralysis, when spinal nerve ligation/injury is successfully induced.

Paw mechanical withdrawal threshold (PWT) was used to measure the mechanical allodynia and was assessed by testing the left hind paw withdrawal response to Von Frey filaments (Stoelting Co., USA) and performed using the up-down method of Dixon [17] at 9:00–12:00 AM. The PWT was calculated using the formula described by Chaplan et al. [18]. The stimulation intensity was determined by one of nine Von Frey filaments (0.407, 0.692, 1.202, 2.041, 3.63, 5.495, 8.511, 11.749, and 15.136 g). A response was considered positive if the animal withdrew its hind paw within 6~8 seconds excluding any body movement. If no response was observed after these five stimulations, a stronger filament was applied. If the rat withdrew its hind paw in response to any of the five stimulations, a filament of the next weaker stimulation level was chosen. The behavioural data were collected before surgery on day 1 before operation for baseline and 3, 7, and 14 days after the operation.

The paw thermal withdrawal latency (PWL) was used to measure the thermal hyperalgesia and was performed using a heat pain stimulator (PL-200, Taimen Biotech Company, Chengdu). The measurement was repeated 5 times for each rat (interval ≥5 min), and the mean was calculated as the PWL for this measurement. All behavioural tests were performed in a double-blind manner.

After carrying out the behavioural assessment, the rats were deeply anesthetised with a chloral hydrate overdose (400 mg/kg). The L4–L6 spinal cord tissue was removed rapidly and fast-frozen in liquid nitrogen until the time of analysis and then rats were killed quickly.

### 2.3. Total RNA and Protein Purification

The total samples were prepared using a DNA/RNA/Protein Isolation Kit (Tiangen Biotech Company, Beijing). The total RNA and proteins were all column purified as a separation matrix. The purified RNA was eluted with the RNA elution solution and stored at −70°C. After the RNA was eluted from the column, the flow through was loaded back onto the column to bind the proteins. The bound proteins were washed with the wash buffer and stored at −20°C.

### 2.4. Quantitative Real-Time Polymerase Chain Reaction (QPCR)

Two micrograms of each total RNA were used to synthesise the complementary cDNA by reverse transcription with a Transcriptor First-Strand cDNA Synthesis Kit (Roche Applied Science co., Shanghai). Templates were amplified in a 25 *μ*L reaction mixture containing 12.5 *μ*L of Fast Start Universal SYBR Green Master (ROX) (Roche Applied Science co., Shanghai), 9.5 *μ*L of DNase-free water, 1 *μ*L each of forward and reverse primer, and 1 *μ*L of cDNA solution. The sequences of forward and reverse primers for HMGB1, TLR4, and *β*-actin genes are shown in Table 1.

Quantitative real-time polymerase chain reaction (QPCR) was performed with the CF × 96 Touch Real-Time PCR system (Bio-Rad Company, US) with the following amplification conditions: 95°C for 3 minutes, followed by 44 cycles of 15 seconds at 95°C and 1 minute at 55°C, a melt curve of 65°C to 95°C, and an increment 0.5°C for 5 seconds. The cDNA amount in each sample was normalised to the crossing point of the housekeeping gene *β*-actin. All experiments were repeated twice, and, in each experiment, PCR reactions were performed in triplicate. HMGB1 and TLR4 gene quantities were estimated from the threshold amplification cycle number (Ct) using Bio-Rad CFX Manager software (Bio-Rad Company, US). The Ct values of both HMGB1 and TLR4 gene are normalised to the reference gene *β*-actin.

Normalised expression is a convenient way to analyse the relative changes in gene expression from real-time quantitative PCR experiments. Normalised expression is also known as the 2^–ΔΔCt^ method, where ΔΔCt = ΔCt sample −  ΔCt control. ΔCT value of HMGB1 and TLR4 was calculated for each sample by subtracting their Ct value from the Ct value for the corresponding *β*-actin. The ΔΔCt values of HMGB1 and TLR4 quantities were calculated with the following formula: ΔΔCt = (CT, HMGB1/TLR4-CT, *β*-actin) Time *x*  − (CT, HMGB1/TLR4-CT, *β*-actin) Time 0. The mean CT values for both HMGB1/TLR4 and *β*-actin were determined at time zero. *β*-actin served as an endogenous internal standard control for variations in RT-PCR.

### 2.5. Western Blotting Analysis

The protein concentrations were estimated using a bicinchoninic acid (BCA) protein assay kit (Tiangen Biotech Company, Beijing). The sample was heated to 95°C for 5 minutes and separated by 10% SDS polyacrylamide gel electrophoresis (Bio-Rad Laboratories, US) and transferred to polyvinylidene difluoride membranes (PVDF, Solarbio Science and Technology Company, Beijing) by electroblotting (Bio-Rad Company, US). Then, the protein was transferred onto a membrane blocked with 5% nonfat dry milk for 2 hours and incubated overnight with primary antibodies (anti-HMGB1 mouse IgG, 1 : 10000; anti-TLR4 mouse IgG, 1 : 5000; anti-*β*-actin mouse IgG 1 : 1000, Abcam, Hong Kong); *β*-actin was used as an internal control.

After incubation with horseradish peroxidase- (HRP-) conjugated secondary antibody (goat polyclonal to mouse IgG, 1 : 30000, Abcam, Hong Kong) for 2 hours, the protein was detected by enhanced chemiluminescence (ECL) (Abcam, Hong Kong). The densities of the protein blots were analysed using image software (USA). Target protein levels were normalised against the *β*-actin level and expressed as relative fold changes. The intensity of blots was quantified with densitometry by personnel blinded to the different treatments.

### 2.6. Enzyme-Linked Immunosorbent Assay (ELISA)

Proinflammatory cytokine (TNF-*α*, IL-1*β*, and IL-10) expression in the spinal cord was analysed using ELISA and measured using rat ELISA kits (Yuanye Biotech Company, Shanghai). All assays were performed according to the instructions of the manufacturers. The absorbance (A) was detected at 450 nm (A450), and the standard curve was delineated based on the A of standards by plotting the mean A value for each standard on the *y*-axis against the concentration on the *x*-axis and drawing a best fit curve through the points on the graph. The standard equation was generated using Curve Expert software. A standard curve and equation were used to determine the amount in an unknown sample.

### 2.7. Statistical Analysis

The data are expressed as the mean ± standard error of the mean (SEM) for the RT-PCR analysis. The normalised expression (2^–ΔΔCt^ method) was employed to calculate the normalised expression of each gene for each sample using Bio-Rad optical system software (Bio-Rad Company, US). All of the data were analysed using nonparametric Kruskal-Wallis test followed by the Mann-Whitney *U* test by SPSS 13.0 statistical software. A difference was accepted as significant if *P* < 0.05.

## 3. Results

### 3.1. Behavioural Assessment of SNL Rats for Validation of Hyperalgesia

The PWT in the SNL group was lower postoperatively on the 3rd, 7th, and 14th days (*P* < 0.05 versus sham group). In addition, the PWT in the Tan IIA group was higher on the postoperative 7th and 14th days (*P* < 0.05 versus SNL group).

**Figure 1:**
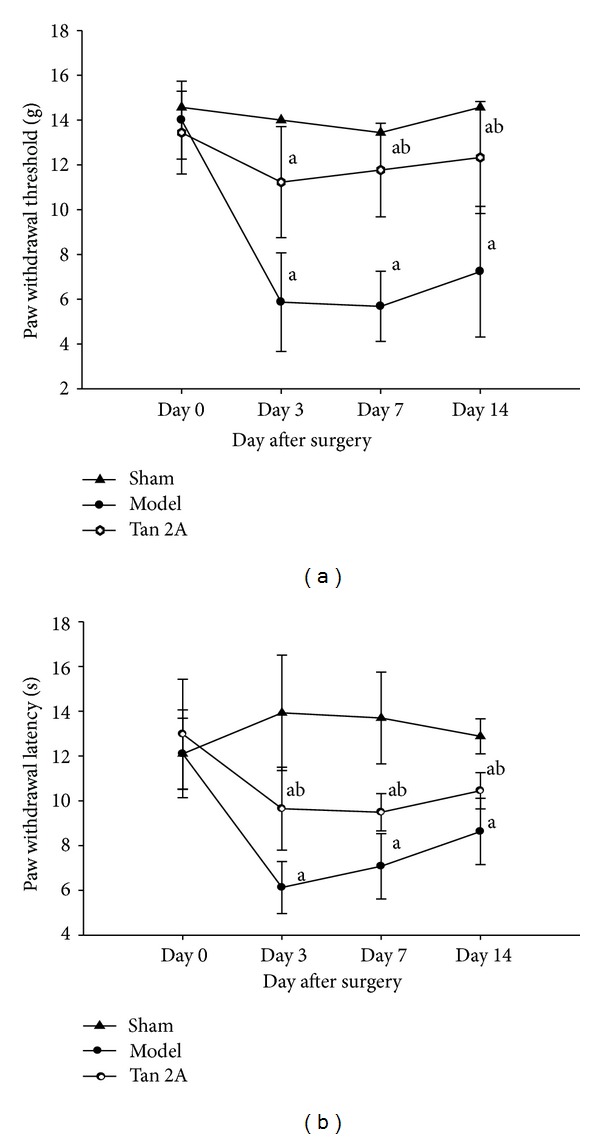
Tanshinone IIA alleviates the neuropathic pain. (a) PWT decreased in the SNL rats (^a^ *P* < 0.05 versus sham group). After Tan IIA treatment, the PWT increased (^b^ *P* < 0.05 versus model group). Data are shown as the mean ± S. D. (b) PWL decreased in the SNL rats (^a^ *P* < 0.05 versus sham group). After Tan IIA treatment, the PWL increased (^b^ *P* < 0.05 versus model group). Data are shown as the mean ± S.D. L: lumbar. SNL: spinal nerve ligation. PWT: paw withdrawal threshold (g). PWL: paw withdrawal latency. S.D: standard deviation.

The PWL remained unchanged in the sham group but was lower postoperatively on the 3rd, 7th, and 14th day in the SNL group (*P* < 0.05 versus sham group), indicating that thermal hyperalgesia was induced by the SNL operation. The PWL in the Tan IIA group increased on the postoperative 7th and 14th days (*P* < 0.05 versus SNL group), indicating that Tanshinone IIA could alleviate the neuropathic pain (Figure 1).

### 3.2. Downregulation of HMGB1 and TLR4 in the Spinal Cord at the mRNA Level

**Figure 2:**
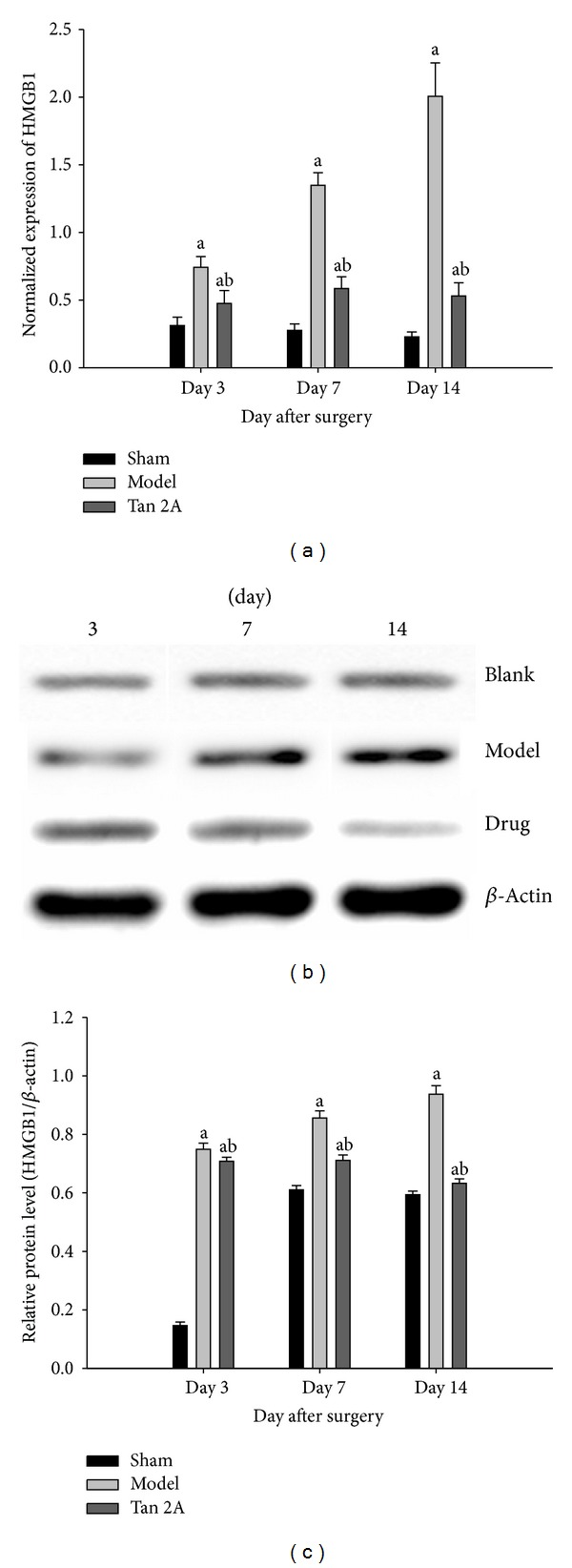
(a) Transcript measured by quantitative real-time PCR showed significant differences in the HMGB1 gene level on the different postoperative days (^a^ *P* < 0.05 versus sham group, ^b^ *P* < 0.05 versus model group). (b) The HMGB1 protein measurement by western blotting showed that the HMGB1 (26 kDa) protein was at a low level in the sham group; the HMGB1 protein began to increase 3 days after SNL surgery and remained at a high level during the experiment (^a^ *P* < 0.05 versus sham group); after Tan IIA treatment, the HMGB1 protein reduced significantly (^b^ *P* < 0.05 versus model group). (c) Summary data of western blot showed significant differences in the HMGB1 protein level on different postoperative days (^a^ *P* < 0.05 versus sham group, ^b^ *P* < 0.05 versus model group). Data are shown as the mean ± S.D. SNL: spinal nerve ligation. HMGB1: high-mobility group box 1. Blank: sham group. Drug: Tan IIA group. S.D: standard deviation. Mean ± S.D.

**Figure 3:**
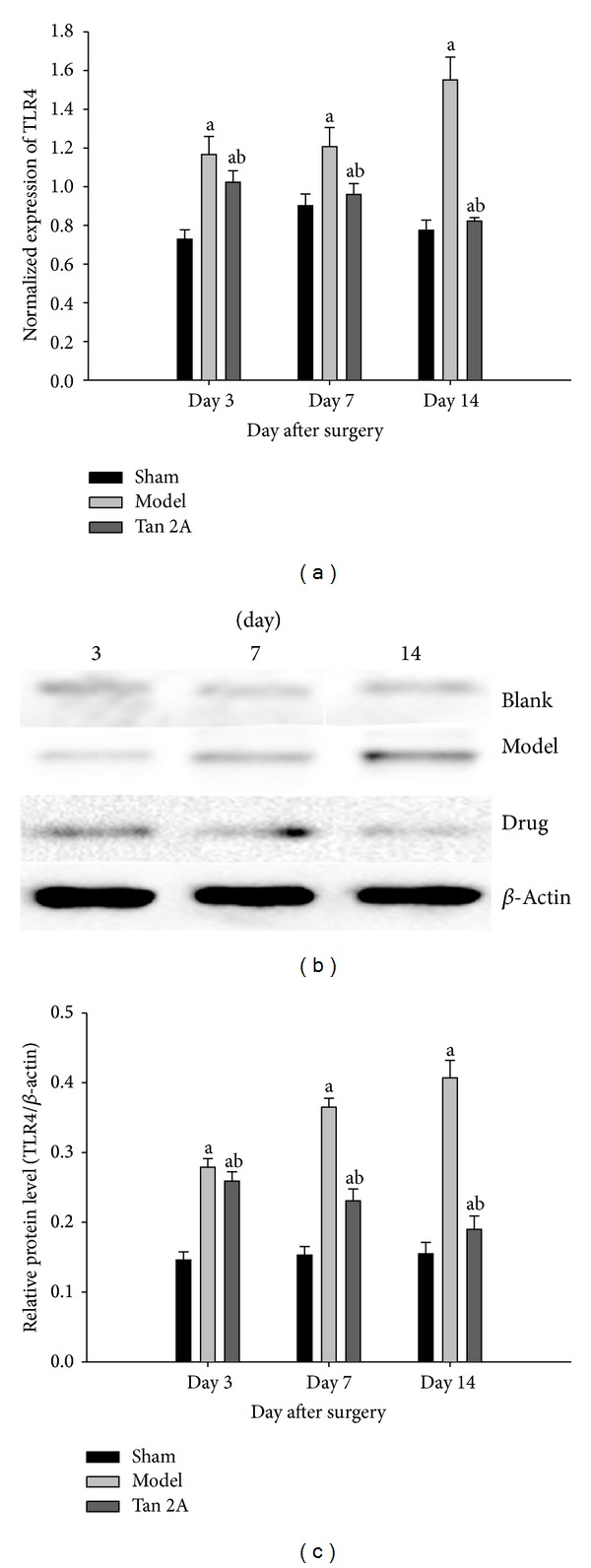
(a) Transcript measured by quantitative real-time PCR showed significant differences in the TLR4 gene level on the different postoperative days (^a^ *P* < 0.05 versus sham group, ^b^ *P* < 0.05 versus model group). (b) The TLR4 measurement by western blotting showed that the TLR4 (94 kDa) protein was at a low level in the sham group; the TLR4 protein began to increase at 3 days after SNL surgery and remained at a high level during the experiment (^a^ *P* < 0.05 versus sham group); after Tan IIA treatment, the TLR4 protein reduced significantly (^b^ *P* < 0.05 versus model group). (c) Summary data of western blot showed significant differences in the TLR4 protein level on different postoperative days (^a^ *P* < 0.05 versus sham group, ^b^ *P* < 0.05 versus model group). Data are shown as the mean ± S.D. SNL: spinal nerve ligation. TLR4: Toll-Like Receptor 4. Blank: sham group. Drug: Tan IIA group. S.D: standard deviation.

The expression of the HMGB1 and TLR4 genes in the spinal cord in the SNL group was lower on the postoperative 3rd, 7th, and 14th days (*P* < 0.05 versus sham group). The HMGB1 and TLR4 gene levels in the spinal cord in the Tan IIA group were higher on the postoperative 3rd, 7th, and 14th days (*P* < 0.05 versus SNL group). Our results showed that the HMGB1 and TLR4 gene levels were significantly upregulated in the SNL rats. The HMGB1 and TLR4 gene levels decreased after Tan IIA treatment (Figures 2 and 3).

### 3.3. Downregulation of TLR4 and HMGB1 in the Spinal Cord at the Protein Level

Western blots revealed that SNL resulted in increased levels of HMGB1 and TLR4 (*P* < 0.05 versus sham group). In addition, there was significant downregulation of HMGB1 and TLR4 in the Tan IIA administration group that was subjected to SNL on the 3rd, 7th, and 14th day after surgery (*P* < 0.05 versus SNL). Our results revealed that HMGB1 and TLR4 protein levels were significantly upregulated in the SNL rats. The expression of HMGB1 and TLR4 protein decreased after Tan IIA treatment (Figures 2 and 3).

### 3.4. Proinflammatory Cytokine (IL-1*β*, TNF-*α*, and IL-10) Expression

**Figure 4:**
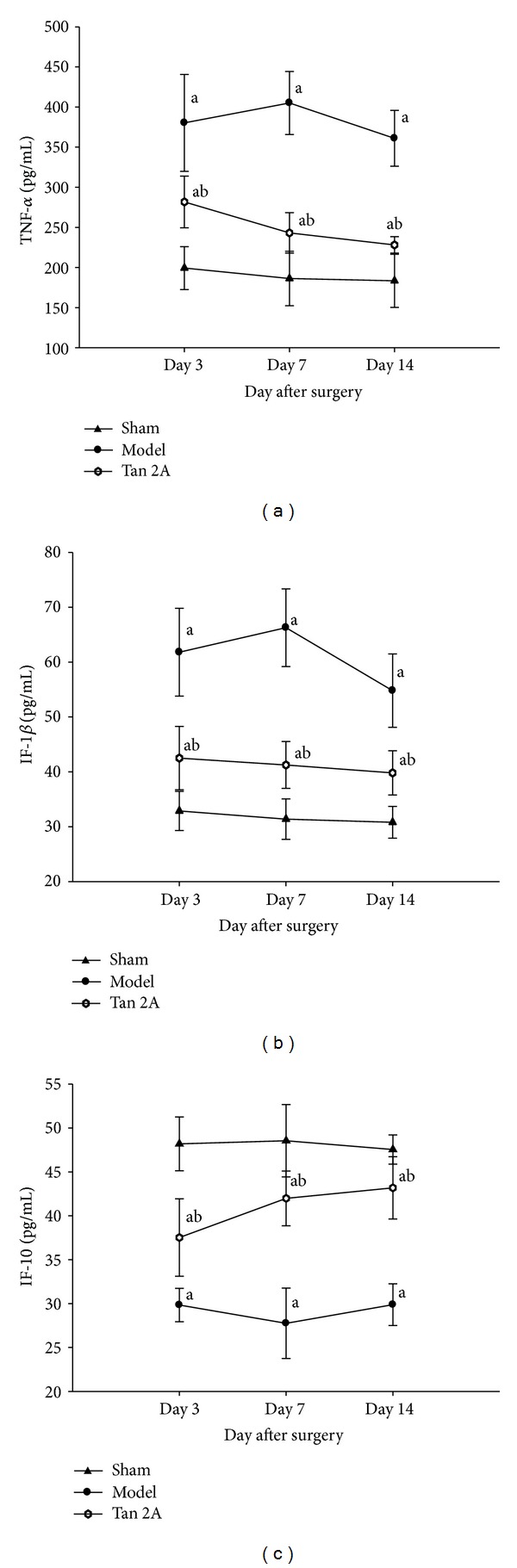
The expression of TNF-*α*, IF-1*β*, and IF-10 in spinal cords of SNL rats on the different postoperative days. (a) TNF-*α* was upregulated in the spinal cord of SNL rats (^a^ *P* < 0.05 versus sham group). After Tan IIA treatment, TNF-*α* expression reduced significantly (^b^ *P* < 0.05 versus model group). (b) IF-1*β* was upregulated in the spinal cord of SNL rats (^a^ *P* <0.05 versus sham group). After Tan IIA treatment, TNF-*α* expression reduced significantly (^b^ *P* < 0.05 versus model group). (c) IF-10 was downregulated in the spinal cord of SNL rats (^a^ *P* < 0.05 versus sham group). After Tan IIA treatment, TNF-*α* expression increased significantly (^b^ *P* < 0.05 versus model group). Data are shown as the mean ± S.D. SNL: spinal nerve ligation. TNF-*α*: tumor necrosis factor alpha. IF-1*β*: interleukin-1 beta. IF-10: interleukin-10. S.D standard deviation.

SNL significantly increased TNF-*α* and IL-1*β* expression at 3, 7, and 14 days after SNL (*P* < 0.05, compared with sham group). In addition, there was significant downregulation of TNF-*α* and IL-1*β* in the Tan IIA administration group that was subjected to SNL (*P* < 0.05, compared with SNL). TNF-*α* and IL-1*β* were upregulated, whereas IL-10 was downregulated in the spinal cord of the SNL rats. IL-10 increased after Tan IIA administration (*P* < 0.05). The results indicated that intraperitoneal Tan IIA significantly attenuated IL-1*β* and TNF-*α* expression and enhanced IL-10 expression in the spinal cord of SNL rats (Figure 4).

## 4. Discussion

The present study demonstrated that SNL produced mechanical allodynia and thermal hyperalgesia by activating the HMGB1-TLR4 pathway, which upregulated HMGB1 and TLR4 expression in the spinal cord of SNL-induced rats. Tan IIA prevented the development of neuropathic pain, inhibited the HMGB1-TLR4 pathway, decreased proinflammatory cytokines, such as TNF-*α* and IL-1*β*, and upregulated IF-10 (a protective factor against neuronal damage).

HMGB1 expression increases in rats that suffer from bone cancer pain [19]. Previous studies have demonstrated that HMGB1 induces nerve injury that is reversible by anti-HMGB1 antibody treatment in neuropathic pain, which suggests that it contributes to pain hypersensitivity. As a cytokine mediator of inflammation, HMGB1 has a critical role in neuropathic pain [20]. After cellular damage or injury, HMGB1 can be translocated from the nucleus to the cytoplasm and secreted from neurons in the maintenance of the neuropathic pain state [21]. HMGB1 may prolong persistent pain states in the pathology of chronic pain due to neuroinflammation [22], which is consistent with the present study. Our data revealed the HMGB1 expression was at a low level in the sham group and began to increase at 3 days after SNL surgery and remained at a high level during the experiment. Thus, we speculated that HMGB1 contributed to the development of pain hypersensitivity.

There is nerve injury-induced expression of HMGB1 and TLR4 in neuropathic pain models. HMGB1 neuronal signalling in neuropathic pain may be dependent on either of two receptors for TLR4 and/or advanced glycation end products (RAGE). The present study revealed that TLR4 expression in the spinal cord of SNL rats was at a low level in the sham group, began to increase at 3 days after SNL surgery, and remained at a high level during the experiment. More interestingly, the amount of TLR4 protein was less than that of HMGB1. Thus, we speculated that HMGB1 combined with the RAGE and TLR4 was depleted in rats of SNL-induced neuropathic pain. As another receptor of HMGB1, RAGE was not investigated in our experiment and should be the target of further investigation in the future.

TLR4 signals through adaptor protein pathways such as the myeloid differentiation primary response gene 88 (MyD88) pathway, leading to activation of NF-*κ*B and producing proinflammatory cytokines such as TNF-*α* and IL-1*β* [23]. Our study revealed that activation of TLR4 inhibited downstream cytokines such as TNF-*α* and IL-1*β* in neuropathic pain models, leading to the development and maintenance of neuropathic pain. This is consistent with other recent reports [24, 25]. This may be occurring through downregulation of HMGB1, TLR4, TNF-*α*, and IL-1*β* levels and upregulation of IF-10 expression. Secretion of HMGB1 can activate these cells, resulting in the release of proinflammatory cytokines, such as TNF-*α*, IL-1*β*, and IL-8, which are associated with hyperalgesia and mechanical allodynia in CCI rats [26].

As an inhibitor of HMGB1, Tan IIA can be used to investigate potential attenuation of neuropathic pain in a SNL model. As Tan IIA is not easily absorbed through the intestinal pathway, sodium tanshinone IIA sulfonate (STS), a water-soluble derivative of Tanshinone IIA, was developed to raise the bioavailability. Thus, STS was used to replace Tan IIA in our present study.

As the results show, Tanshinone IIA reversed SNL-induced thermal hyperalgesia and mechanical allodynia. Tanshinone IIA downregulated HMGB1 and TLR4 levels and inhibited the HMGB1-TLR4 pathway. Tanshinone IIA inhibited cytokine, such as TNF-*α* and IL-1*β* expression, but not IF-10 expression in the spinal cord of SNL rats. Collectively, our data provide new insights into an understanding of the molecular mechanisms of Tanshinone IIA inhibited SNL-induced neuropathic pain, suggesting that targeting the HMGB1-TLR4 pathway could serve as the basis for new antinociceptive agents.

Our results indicate that Tanshinone IIA attenuates SNL-induced neuropathic pain, and the mechanism was caused, at least in part, by a blockade in the HMGB1-TLR4 pathway, presumably through the downregulation of HMGB1-TLR4 signalling, which plays a role in SNL-induced neuropathic pain.

## Figures and Tables

**Table 1:** Primers sequence for the genes characterised in the present experiment.

Genes (length)	Primers	Sequences
HMGB1 (218 bp)	Forward primer	5′ AGC AAT CTG AAC GTC TGT CC 3′
	Reverse primer	5′ GTT CTT GTG ATA GCC TTC GC 3′

TLR4 (356 bp)	Forward primer	5′ GCC GGA AAG TTA TTG TGG TGG T 3′
	Reverse primer	5′ ATG GGT TTT AGG CGC AGA GTT T 3′

*β*-Actin (372 bp)	Forward primer	5′ GCC ATG TAC GTA GCC ATC CA 3′
	Reverse primer	5′ GAA CCG CTC ATT GCC GAT AG 3′
